# Potent bace-1 inhibitor design using pharmacophore modeling, *in silico* screening and molecular docking studies

**DOI:** 10.1186/1471-2105-12-S1-S28

**Published:** 2011-02-15

**Authors:** Shalini John, Sundarapandian Thangapandian, Sugunadevi Sakkiah, Keun Woo Lee

**Affiliations:** 1 Department of Biochemistry and Division of Applied Life Science (BK21 Program), Environmental Biotechnology National Core Research Center (EB-NCRC), Gyeongsang National University (GNU), 900 Gazwa-dong, Jinju 660-701. Republic of Korea

## Abstract

**Background:**

Beta-site amyloid precursor protein cleaving enzyme (BACE-1) is a single-membrane protein belongs to the aspartyl protease class of catabolic enzymes. This enzyme involved in the processing of the amyloid precursor protein (APP). The cleavage of APP by BACE-1 is the rate-limiting step in the amyloid cascade leading to the production of two peptide fragments Aβ_40_ and Aβ_42_. Among two peptide fragments Aβ_42_ is the primary species thought to be responsible for the neurotoxicity and amyloid plaque formation that lead to memory and cognitive defects in Alzheimer’s disease (AD). AD is a ravaging neurodegenerative disorder for which no disease-modifying treatment is currently available. Inhibition of BACE-1 is expected to stop amyloid plaque formation and emerged as an interesting and attractive therapeutic target for AD.

**Methods:**

Ligand-based computational approach was used to identify the molecular chemical features required for the inhibition of BACE-1 enzyme. A training set of 20 compounds with known experimental activity was used to generate pharmacophore hypotheses using *3D QSAR Pharmacophore Generation* module available in Discovery studio. The hypothesis was validated by four different methods and the best hypothesis was utilized in database screening of four chemical databases like Maybridge, Chembridge, NCI and Asinex. The retrieved hit compounds were subjected to molecular docking study using GOLD 4.1 program.

**Results:**

Among ten generated pharmacophore hypotheses, Hypo 1 was chosen as best pharmacophore hypothesis. Hypo 1 consists of one hydrogen bond donor, one positive ionizable, one ring aromatic and two hydrophobic features with high correlation coefficient of 0.977, highest cost difference of 121.98 bits and lowest RMSD value of 0.804. Hypo 1 was validated using Fischer randomization method, test set with a correlation coefficient of 0.917, leave-one-out method and decoy set with a goodness of hit score of 0.76. The validated Hypo 1 was used as a 3D query in database screening and retrieved 773 compounds with the estimated activity value <100 nM. These hits were docked into the active site of BACE-1 and further refined based on molecular interactions with the essential amino acids and good GOLD fitness score.

**Conclusion:**

The best pharmacophore hypothesis, Hypo 1, with high predictive ability contains chemical features required for the effective inhibition of BACE-1. Using Hypo 1, we have identified two compounds with diverse chemical scaffolds as potential virtual leads which, as such or upon further optimization, can be used in the designing of new BACE-1 inhibitors.

## Background

Beta-site amyloid precursor protein cleaving enzyme (BACE-1), also known as β-secretase, memapsin-2, or Aspartyl protease-2, is a single-membrane protein belongs to the aspartyl protease class of catabolic enzyme. This is one of the enzymes responsible for the sequential proteolysis of amyloid precursor protein (APP) [1]. The cleavage of APP by BACE-1, which is the rate-limiting step in the amyloid cascade, results in the generation of two peptide fragments Aβ40 and Aβ42. Among two peptide fragments, Aβ42 is the primary species and thought to be causal for the neurotoxicity and amyloid plaque formation that lead to memory and cognitive defects in Alzheimer’s disease (AD) [2]. The AD is a debilitating neurodegenerative disease that results in the irreversible loss of neurons, particularly in the cortex and hippocampus [3]. It is characterized by progressive decline in cognitive function that inevitably leading to incapacitation and death. It also histopathologically characterized by the presence of amyloid plaques and neurofibrillar tangles in the brain. Regardless of the increasing demand for medication, no truly disease-modifying treatment is currently available [4,5]. The BACE knockout study in mice shows a complete absence of Aβ production with no reported side effects [6-8]. Since gene knockout study showed a reduction in AD-like pathology, inhibition of BACE-1 the key enzyme in the production of Aβ peptide has emerged as an attractive therapeutic target for AD [9]. Therefore extensive efforts have been followed in the discovery of potential inhibitors of BACE-1. Most of the designing of BACE-1 inhibitors are based on the transition state mimetic approach, which depends mainly on replacing the scissile amide bond of an appropriate substrate with a stable mimetic of the putative transition-state structure [10].

The main aim of our approach, which is discussed in this study is different than the transition state mimetic approach, is to develop an accurate and efficient method for discovering potent BACE-1 inhibitors. A pharmacophore hypothesis was generated based on key structural features of compounds with BACE-1 inhibitory activity. It provides a rational hypothetical representation of the most important chemical features responsible for activity. Herein, a ligand-based 3D pharmacophore hypothesis for BACE-1 inhibitors was constructed based on the structure-activity relationship observed in a set of known BACE-1 inhibitors. The resulted pharmacophore hypotheses were validated by test set, Fischer randomization, leave-one-out, and decoy set methods. The validated pharmacophore hypothesis has been used in *in silico* screening to identify hits that are highly varied in chemical nature. The retrieved hits were subsequently subjected to a well-defined refining procedure based on estimated activity values, drug-likeness prediction and further by molecular docking study. The identified hits can further be utilized in designing novel and potent BACE-1 inhibitors.

## Methods

### Dataset collection

In a computerized pharmacophore generation process the accurate choice of the training set is a key issue. The built pharmacophore hypothesis can be as good as the input data information. The following criteria should be considered during the selection of data set in order to achieve a significant pharmacophore hypothesis. (1) All compounds used in the training set have to bind to the same receptor in roughly the same fashion. Compounds having more binding interaction with the receptor are more active than those with fewer; (2) the data set must be widely populated covering an activity range of at least 4 orders of magnitude; (3) the most active compounds should inevitably be included in the training set and (4) all biologically relevant data should be obtained by homogenous procedures [11]. Every individual feature in the resulting hypotheses will invade a certain weight that is proportional to its relative contribution to biological activity.

**Figure 1:**
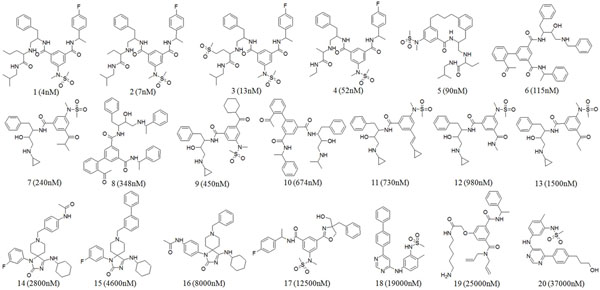
**Structure of training set compounds.** The 2D chemical structures of 20 compounds of the training set together with their experimental IC_50_ values in nM.

By taking these criteria into account, we have collected a total number of 320 BACE-1 inhibitors from various literature resources [12-25] and a database has been created. The 2D structure of the compounds were built using ChemSketch program version 12, and subsequently converted in to 3D structures using Discovery studio 2.5 (DS) [26]. In the next step, 60 compounds were selected as final dataset as the BACE-1 inhibitory activities of these 60 compounds were studied under same biological assay condition. Based on the principle of structural diversity and wide coverage of activity range, 20 compounds were carefully selected as training set compounds and the rest were used as test set in model validation. Here, the IC_50_ value of the training set compounds was taken into account, the inhibitory activity values of the training set compounds span over a range of four orders of magnitude, from 4 nM to 37 000 nM. The chemical structure and experimental activity of the training set compounds are shown in Figure 1.

### Diverse conformation generation

Prior to the generation of pharmacophore hypotheses, the training set compounds, which were converted to 3D structure, were used to generate diverse conformations. *Diverse Conformation Generation* protocol implemented in DS was used to generate conformations using the *Best* conformation model generation method with CHARMM force field and Poling algorithm to ensure the energy-minimized conformation for each compound. The parameters like maximum number of 250 conformers, the ‘best conformational analysis’ method, and an energy threshold of 20 kcal/mol above the global energy minimum were chosen during conformation generation.

### Pharmacophore modeling

The training set comprises of 20 compounds was used in pharmacophore hypothesis generation. The HypoGen algorithm available in *3D QSAR Pharmacophore Generation* protocol of DS tries to generate hypotheses with features common amongst active molecules and do not reflect the inactive molecules of the training set. The training set compounds were predicted for their inherent chemical features using *Feature Mapping* protocol implemented in DS. During pharmacophore hypothesis generation a minimum of 4 and a maximum of 5 pharmacophoric features like hydrogen bond acceptor (HBA), hydrogen bond donor (HBD), positive ionizable (PI), ring aromatic (RA) and hydrophobic (HY) were included. These features were selected based on the feature mapping results. All parameters were set to their default values except uncertainty value, which has been changed to 2 instead of 3. An uncertainty value of 2 was more convenient for our dataset because the activity values of the training set spanned exactly the required 4 orders of magnitude; this choice has been further confirmed by preliminary calculations and by other literature evidence [27]. The uncertainty value represents the ratio of the uncertainty range of the actual activity against measured biology activity for each compound.

### The HypoGen algorithm

With the full range of training set compounds from active to inactive the pharmacophore hypotheses were generated by HypoGen algorithm implemented in DS. This algorithm constructs and ranks the pharmacophore hypotheses that correlate best between 3D spatial arrangement of features in a given training set compounds and their respective experimental activities. This process is accomplished in three steps: the constructive phase, the subtractive phase and the optimization phase [28].

The constructive phase identifies the hypotheses that are common amongst the active compounds. HypoGen enumerates all possible pharmacophore configurations using all combinations of pharmacophore features for each of the conformations of the most active compound. In order to consider the left over most active compounds the hypotheses must fit a minimum subset of its features. Hence, a large database of pharmacophore configurations will be generated at the end of the constructive phase.

The subtractive phase will remove the pharmacophore configurations that are present in the least active compounds. All compounds whose activity is by default 3.5 orders of magnitude less than that of the most active compound are considered to represent the least active compounds. The value 3.5 is adjustable depending on the activity range of the training set. The optimization phase improves the hypothesis score. These scores of the generated hypotheses depend on the errors in activity estimation from regression and complexity. The optimization involves a variation of features and/or locations to optimize activity prediction via a simulated annealing approach. The total cost parameter will be calculated for every new hypothesis. The HypoGen will quit and reports the 10 top-scoring hypotheses when there is no improvement in the hypothesis score.

### Data analysis

The quality of a pharmacophore hypothesis is best determined by two theoretical cost calculations, which are represented in bit units [29]. One is the ‘fixed cost’ also known as cost of an ideal hypothesis, which represents the simplest model that fits all the data perfectly. The second cost is the ‘null cost’, which represents the highest cost of a pharmacophore with no features that estimates every activity to be the average of the activity data of the training set compounds.

The total cost of any pharmacophore hypothesis should always be close to the fixed cost and away from the null cost to be the significant model. The cost difference between fixed and null cost values should be larger for a meaningful pharmacophore hypothesis. A value of 40-60 bits in a pharmacophore hypothesis indicates that it has 75-90% probability of representing a true correlation in the data.

The hypotheses are also evaluated based on other cost components. The cost value for every individual hypothesis is the summation of three cost components: the error cost (E), the weight cost (W) and the configuration cost (C). The error cost is the value represents the root-mean-squared difference (RMSD) between experimental and estimated activity value of the training set compounds. The weight cost is a value that increases in a Gaussian form as this function weights in a model deviate from the ideal value of two. The configuration cost or entropy cost measures the entropy of the hypothesis space. If the input training set compounds are too multiplex, e.g. because of too flexible training set compounds, this will result in an effusive number of hypotheses as an outcome of the subtractive phase. This configuration cost should always be less than a maximum value of 17 [30]. The correlation coefficient of the pharmacophore hypothesis should be close to 1.

### Pharmacophore validation

The generated pharmacophore hypothesis was validated using test set, Fischer randomization, decoy set and leave-one-out methods.

#### Test set method

A total of 40 compounds with experimental activity data were selected as test set compounds. This method is used to elucidate whether the generated pharmacophore hypothesis is proficient to predict the activities of the compounds other than training set and classify them correctly in their activity scale. The conformation generation for test set compounds was carried out in a similar way like training set compounds using *Diverse Conformation Generation* protocol in DS. The compounds associated with their conformations were subsequently carried out for pharmacophore mapping using *Ligand Pharmacophore Mapping* protocol with *Best/Flexible Search* option available in DS.

#### Fischer randomization method

The main purpose of this validation is to verify whether there is a strong correlation existing between the chemical structure and biological activities of compounds. This generates pharmacophore hypotheses by randomizing the activity data of the training set compounds with the same features and parameters used to generate the original pharmacophore hypothesis. The statistical significance is calculated using the following formula: Significance = 100 (1-(1+x/y)), where *x* represents the total number of hypotheses having a total cost value lower than the original hypothesis, and *y* represents the total number of HypoGen runs i.e. initial and random runs. The confidence level was set to 99%, where 99 random spreadsheets (random hypotheses) were generated. During the pharmacophore generation, if the randomized data set results in similar or better cost values, RMSD and correlation, it means that the original hypothesis have been generated by chance.

#### Decoy set method

An external database containing BACE-1 active and inactive compounds was used to evaluate the discriminative ability of Hypo 1 in the separation of active compounds from the inactive compounds. The database was developed using a total of 453 compounds containing 206 actives and 247 inactives. All the compounds were collected from published literature including binding database [12-25,31]. The database screening was carried out using *Ligand Pharmacophore Mapping* protocol available in DS. A set of statistical parameters [32] like Ht, % yield of actives, Enrichment factor (E), false positives, false negatives and Goodness of Hit (GH) score were calculated.

#### Leave-one-out method

The pharmacophore hypothesis is cross validated by leave-one-out method. In this method, one compound is left in the generation of a new pharmacophore model and its affinity is predicted using that new model. The model building and estimation cycle is repeated until each compound was left out once [33]. This test is performed to verify whether the correlation coefficient of the training set compounds is strongly depend on one particular compound or not [34].

### Database screening

The validated pharmacophore hypothesis, Hypo 1, was used as a 3D query for screening four different chemical databases. The purpose of this screening is to retrieve novel and potential leads suitable for further development. The chemical databases used were Maybridge, Chembridge, NCI and Asinex. Conformers were generated for each molecule in the database using best conformer generation method that allows a maximum energy of 15 kcal/mol above that of the most stable conformation. The database screening was carried out using *Ligand Pharmacophore Mapping* protocol implemented in DS with *Best/Flexible Search* option. The retrieved compounds were filtered by restricting the estimated activity value less than 100 nM and the obtained compounds were further refined using molecular docking study.

### Molecular docking

Pharmacophore modeling normally firmly associated with docking procedure, which in a first step flexibly aligns the ligand molecule into a rigid macromolecule environment and then estimates the tightness of the interaction by different scoring functions [35]. The Docking takes all the information from a rigid protein environment and scores several possible interaction modes for different alignments. There are many docking programs available for molecular docking studies. In this study, we used GOLD (Genetic Optimisation for Ligand Docking), a docking program [36] that uses genetic algorithm for docking and performs automated docking with full acyclic ligand flexibility, partial cyclic ligand flexibility and partial protein flexibility in the neighborhood of the protein active site. The crystal structure of BACE-1 complexed with an inhibitor SC7 (PDB ID: 2QP8) was used in molecular docking studies. The inhibitor SC7 was extracted from the active site and the retrieved database hits were docked based on the ligand SC7 coordinates, in to the active site of BACE-1. The water molecules were removed prior to docking because they were not found to play any important roles in BACE1-ligand interaction. The early termination option parameter in GOLD was changed from 3 to 5 and the maximum save conformations was set to 10. All the other parameters were set at their default values.

## Results and discussion

### Pharmacophore generation

We have used the HypoGen algorithm implemented in DS in order to quantitatively correlate the chemical structure of BACE-1 inhibitors to their biological activity. The training set of 20 compounds (Figure 1) with activity values ranging from 4 to 37000 nM was used in pharmacophore model generation. The *Feature Mapping* protocol resulted in HBA, HBD, RA, PI and HY features. Selecting these features, the pharmacophore generation run was performed along with diverse conformers of training set molecules generated as described in methods section. Ten top-scored pharmacophore hypotheses were generated and in order to choose the best one and also to give an idea about the statistical significance, the pharmacophore hypotheses were subjected to cost analysis. The results of top ten pharmacophore hypotheses and their statistical parameters are given in Table 1. In this study, the first pharmacophore hypothesis (Hypo 1) is the best hypothesis characterized by the large cost difference (121.98 bits), lowest RMSD value (0.804) and a high correlation coefficient of 0.977. All ten hypotheses consist of HBD, PI, RA and HY features. Nine of ten hypotheses were composed of five pharmacophoric features except only one hypothesis, which was of four features. The best pharmacophore hypothesis (Hypo 1), which scored the large cost difference, lowest RMSD, lowest error cost and high correlation coefficient, was made of one HBD, one PI, one RA and two HY features.

**Table 1:** Results of the top 10 pharmacophore hypotheses generated by the *HypoGen* algorithm

Hypothesis	Total cost	Cost difference^a^	RMSD	Corr. (r)	Features^b^	Test set correlation coefficient
Hypo 1	81.24	121.98	0.804	0.977	HBD, PI, RA, HY, HY	0.917
Hypo 2	81.44	121.78	0.813	0.976	HBD, PI, RA, HY, HY	0.902
Hypo 3	81.80	121.42	0.836	0.975	HBD, PI, RA, HY, HY	0.882
Hypo 4	82.13	121.09	0.852	0.974	HBD, PI, RA, HY, HY	0.861
Hypo 5	83.00	120.22	0.907	0.971	HBD, PI, RA, HY, HY	0.899
Hypo 6	85.49	117.73	1.035	0.962	HBD, PI, RA, HY, HY	0.901
Hypo 7	86.57	116.65	1.082	0.959	HBD, PI, RA, HY, HY	0.872
Hypo 8	86.75	116.47	1.085	0.958	HBD, PI, RA, HY	0.859
Hypo 9	86.87	116.35	1.098	0.957	HBD, PI, RA, HY, HY	0.862
Hypo 10	87.53	115.9	1.111	0.956	HBD, PI, RA, HY, HY	0.875

Null cost = 203.22; fixed cost = 74.77; configuration cost = 15.59.

^a^Cost difference = null cost – total cost.

^b^HBD, hydrogen-bond donor; PI, positive ionizable; RA, ring aromatic; HY, hydrophobic.

### Statistical data analysis

**Figure 2:**
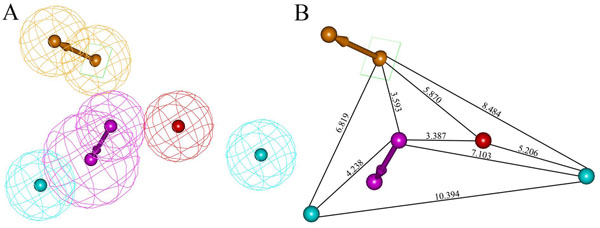
**HypoGen pharmacophore hypothesis for BACE-1 inhibitors.** A) The best five feature pharmacophore model Hypo 1 B) 3D spatial arrangement and the distance constraints of the Hypo 1. The features are color coded with magenta, hydrogen bond donor; red, positive ionizable; orange, ring aromatic; cyan, hydrophobic features.

The generated hypotheses were subjected to cost analysis. The two main values used for cost analysis are the difference between fixed and null cost and another is the difference between the null cost and the total cost (Δcost). The fixed cost of the run was 74.77 bits, which was well separated from the null cost of 203.22 bits and close to the total cost of 81.24 bits. The large difference (128.45 bits) observed between the fixed cost and null cost value indicates that Hypo 1 has more than 90% statistical significance to be a significant model. All the 10 hypotheses were subjected to further evaluation for their capability to predict the activity of the training set compounds. Configuration cost or entropy value must be less than 17 for which a value of 15.59 was obtained in this study. All hypotheses have scored RMSD values lower than 1.5 and ranging from 0.804 to 1.111, this characterization further emphasizing the good predictive quality of these hypotheses. Based on the rule to select a hypothesis with a lowest total cost, high correlation coefficient, large cost difference and significantly low RMSD value, Hypo 1 gave the best statistical values among other hypotheses. Hence, Hypo 1 with one HBD, one PI, one RA and two HY was chosen as the best hypothesis for further analysis. The inter-feature distance constraints were observed for this five-featured pharmacophore hypothesis (Hypo 1) (Figure 2).

### Activity prediction and mapping of training set compound on Hypo1

**Figure 3:**
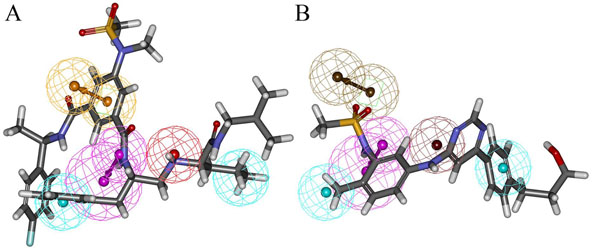
**Pharmacophore mapping of most and least active compounds in the training set.** A) Hypo 1 mapped on to the most active Compound 1 B) Hypo 1 mapped on to the least active Compound 20. The features are color coded with magenta, hydrogen bond donor; red, positive ionizable; orange, ring aromatic; cyan, hydrophobic features.

To verify the predictive ability of Hypo1 on training set compounds, the activity of each training set compound is estimated by regression analysis. The experimental activities of training set compounds were classified into four groups: most active (IC_50_ ≤ 100 nM, ++++), active (100 nM < IC_50_ ≤ 1000 nM, +++), moderately active (1000 nM < IC_50_ ≤ 10 000 nM, ++) and inactive (IC_50_ > 10 000 nM, +). The estimated activity values of training set compounds based on Hypo 1 and the corresponding error values are calculated (Table 2). The error value is the ratio between the estimated and experimental activities. The positive error value indicates that the estimated IC_50_ value is higher than the experimental activity, whereas the negative error value indicates that the estimated IC_50_ value is lower than the experimental activity. An error value of less than 10 signifies the prediction of activity lesser than one order of magnitude. Among 20 training set compounds, only one compound had an error value of greater than 3. From Table 2 it is clear that the estimated activity values of most of the training set compounds was predicted with the same activity scale as the experimental activity. Among 20 training set compounds, one most active compound (++++) was estimated as active (+++), one active compound (+++) was estimated as moderately active (++), one moderately active compound (++) was estimated as inactive (+) and two inactive compounds (+) were estimated as moderately active (++). The divergence between the estimated and experimental activity observed in four compounds was only about 1 order of magnitude, which might be an artifact of the program that uses different number of degrees of freedom for these compounds to mismatch the pharmacophore model. Interestingly, for feature fitting, the most active compounds in the training set mapped well on all the chemical features that are one HBD, one PI, one RA and two HY features of Hypo 1 with good fitting score. The active, moderately and inactive compounds have missed at least one of five features. In addition, the most active compounds mapped well on the RA and PI features whereas some active, moderately active and all inactive compounds could not map on the RA and PI features signifying the importance of these two features. The pharmacophore overlay of most active Compound 1 and Hypo 1 has shown a fit value of 9.52. The RA feature corresponds to phenyl ring present in between two amide and a sulfonamide groups, one HBD feature corresponds to nitrogen of amide group located at the branch, PI group corresponds to the only amino nitrogen, one HY feature corresponds to a phenyl group whereas the another HY feature corresponds to alkyl group (Figure 3A). The pharmacophore overlay of least active compound 20 has revealed that it missed two features when mapped on Hypo 1 with a fit value of 5.16. This compound has mapped only the HBD and two HY features in the same manner as most active compound with no mapping over RA and PI features (Figure 3B). Fit value indicates how well the features in the pharmacophore overlaps the chemical features in the molecule and thereby aid in understanding the chemical meaning of the hypothesis [37]. These results emphasized Hypo 1 as a reliable model to accurately estimate the experimental activity of the training set compounds.

**Table 2:** Experimental and estimated IC_50_ values of the training set compounds based on the pharmacophore hypothesis ‘Hypo 1’.

Compound	IC_50_ nM		Error^a^	Fit value^b^	Activity scale^c^	
	
	Experimental	Estimated			Experimental	Estimated
1	4	1.7	-2.4	9.52	++++	++++
2	7	16	+2.3	8.54	++++	++++
3	13	13	-1	8.64	++++	++++
4	52	88	+1.7	7.80	++++	++++
5	90	150	+1.7	7.57	++++	+++
6	115	170	+1.5	7.51	+++	+++
7	240	190	-1.3	7.48	+++	+++
8	348	320	-1.1	7.24	+++	+++
9	450	470	+1.1	7.07	+++	+++
10	674	460	-1.5	7.08	+++	+++
11	730	610	-1.2	6.97	+++	+++
12	980	1400	+1.4	6.60	+++	++
13	1500	880	-1.7	6.80	++	+
14	2800	8100	+2.9	5.84	++	++
15	4600	5200	+1.1	6.03	++	++
16	8000	10000	+1.3	5.73	++	++
17	12000	8000	-1.6	5.84	+	++
18	19000	19000	-1	5.48	+	+
19	25000	6100	-4.1	5.96	+	++
20	37000	38000	+1	5.16	+	+

^a^Positive value indicates that the estimated IC_50_ is higher than the experimental IC_50_; negative value indicates that the estimated IC_50_ is lower than the experimental IC_50_.

^b^Fit value indicates how well the features in the pharmacophore map the chemical features in the compound.

^c^Activity scale: most active, ++++, IC_50_ ≤ 100 nM; active, +++, 100 nM < IC_50_ ≤ 1000 nM; moderately active, ++, 1000 nM < IC_50_ ≤ 10,000 nM; inactive, +, IC_50_ > 10,000 nM.

### Validation of Hypo 1

Hypo 1 was further validated by test set, Fischer randomization test, leave-one-out and decoy set methods.

#### Test set method

**Figure 4:**
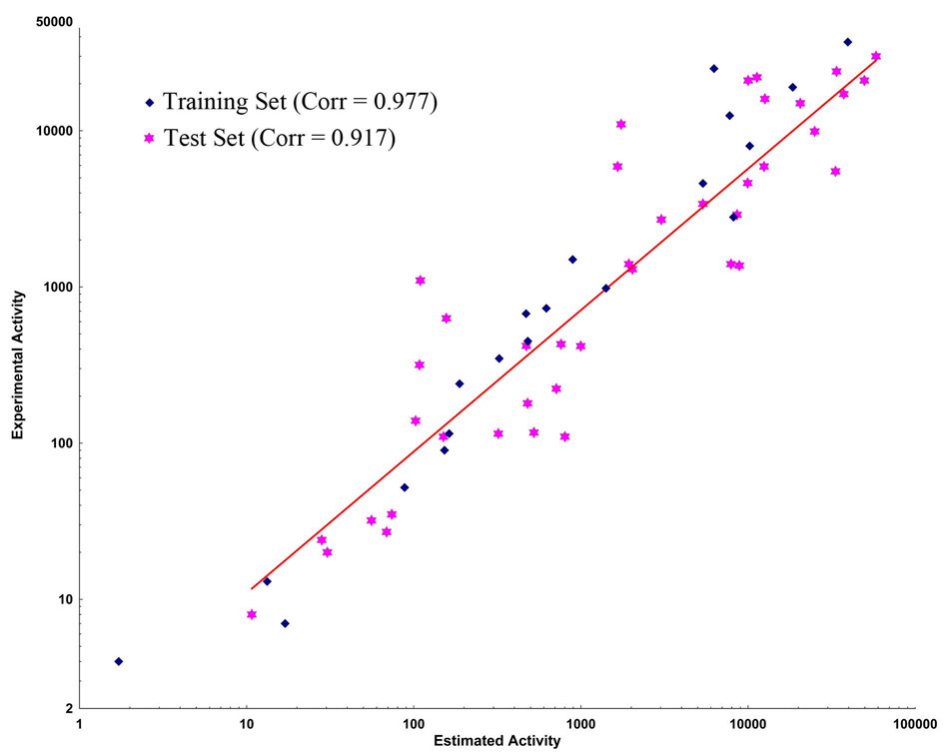
**Test set correlation graph.** Graph showing the correlation between experimental and Hypo 1 estimated activities of the 40 test set compounds along with 20 training set compounds.

A total of 40 compounds structurally different from the training set compounds were selected as test set. The test set compounds were prepared in the same way training set compounds were prepared. The top-scored 10 hypotheses was regressed against 40 test set compounds and calculated the correlation coefficient values ranging from 0.917 to 0.859 (Table 1) between experimental and estimated activities. Among 10 hypotheses, Hypo 1 has given a correlation coefficient of 0.917 (Figure 4) indicating a good correlation between the estimated and experimental activities. The predictive ability of the Hypo 1 against test set compounds was considered better than other hypotheses and the estimated activity values along with the experimental and error values based on Hypo 1 are tabulated (See additional file 1: Experimental and estimated IC50 values of the test set compounds based on the pharmacophore hypothesis ‘Hypo 1’.). Most of the test set compounds was estimated correctly to their experimental activity. The test compounds were classified into four groups in a similar way as that of training set: most active (IC_50_ ≤ 100 nM, ++++), active (100 nM < IC_50_ ≤ 1000 nM, +++), moderately active (1000 nM < IC_50_ ≤ 10 000 nM, ++) and inactive (IC_50_ > 10 000 nM, +). A total of 11 out of 12 active (+++) compounds were estimated correctly as active, but 1 compound was estimated as most active (++++). Interestingly all the six most active (++++) compounds were estimated correctly as most active (++++). Total of twelve active (+++) compounds were estimated correctly as active. Out of thirteen moderately active (++) compounds only one compound was over estimated as active (+++), and 3 compounds were under estimated as inactive (+), whereas nine compounds were estimated correctly as moderately active. Among nine inactive (+) compounds one was over estimated as moderately active (++) compound whereas eight were estimated as inactive compounds. A total of 2 inactive (+) compounds out of 9 were over estimated as moderately active (++) whereas 7 were correctly estimated as inactive compounds. These results suggested that Hypo 1 has a good agreement with the experimental data and able to predict the activities of a wide variety of BACE-1 inhibitors.

#### Fisher randomization method

This method is used to evaluate the statistical significance of Hypo 1 based on the principle of randomizing the activity data of the training set compounds. During validation process random spreadsheets were generated using the training set molecules, and randomly reassigns the activity values to each compound. Subsequently generates the pharmacophore hypotheses using the same features and parameters used in the development of original hypothesis, Hypo 1. A total of 99 random spreadsheets (random hypotheses) required to be generated to achieve a confidence level of 99%. The results of top 10 random spreadsheets along with Hypo 1 are presented in Table 3. None of the top 99 radomly generated hypotheses has scored a total cost lower than the original hypothesis. The statistics of Hypo 1 is far more superior to the top 10 random hypotheses as well as the other 89 random hypotheses. This cross validation results clearly shows that the Hypo 1 was not generated by chance, and has strong confidence to represent a true correlation in the training set.

**Table 3:** Fischer’s randomization test results of the pharmacophore hypothesis Hypo 1.

Validation no.	Total cost	Fixed cost	RMSD	Correlation
Original hypothesis				
Hypo1	81.24	74.770	0.804	0.977
Randomization results				
Trial1	116.69	66.395	2.232	0.811
Trial2	133.35	69.600	2.496	0.756
Trial3	124.05	68.126	2.372	0.783
Trial4	189.35	62.993	3.500	0.397
Trial5	171.63	68.169	3.211	0.539
Trial6	158.05	63.578	3.074	0.591
Trial7	116.78	64.678	2.191	0.821
Trial8	135.62	68.457	2.580	0.736
Trial9	140.83	69.702	2.667	0.714
Trial10	172.23	64.740	3.257	0.520

#### Decoy set method

The Hypo 1 was further validated using an external database for its ability to select BACE-1 inhibitors. This database contains a total (D) of 453 compounds including 206 active (A) compounds. Using Hypo 1, this database screening was carried out, 230 compounds were retrieved as hits (H_t_). The results of GH score and E-value calculation are given in Table 4. Among 230 retrieved hit compounds, 197 compounds were from known actives (Ha). The false positive value is 33 and the false negative value is 9. The calculated E value was 1.88 indicates that the model is highly efficient for database screening. The GH value is expected to be greater than 0.7, which indicates a good model [38]. It was observed to be 0.76 for Hypo 1 and proving its ability in predicting the active compounds among inactives.

**Table 4:** Statistical parameters of GH score validation for Hypo 1.

S. No	Parameters	Results
1	Total molecules in database (D)	453
2	Total no. of actives in database (A)	206
3	Total hits (Ht)	230
4	Active hits (Ha)	197
5	% Yield of actives [(Ha/Ht) X 100]	85.65
6	% Ratio of actives [(Ha/A) X 100]	95.63
7	Enrichment factor (E) [(Ha X D)/(Ht X A)]	1.88
8	False negatives [A - Ha]	9
9	False positives [Ht - Ha]	33
10	Goodness of hit (GH)*	0.76

*[(Ha/4HtA)(3A + Ht) X (1 – (Ht – Ha)/(D –A))]; GH score of 0.6-0.8 indicates a very good model.

#### Leave-one-out method

The cross validation of the model was done using the leave-one-out method. This method is progressed by recomputing the pharmacophore hypotheses by leaving one compound at a time from the training set compounds. The importance of this validation is to prove that the correlation of the original pharmacophore hypothesis (Hypo 1) is not depending only on one particular compound. If the activity of each left-out compound is correctly estimated by the corresponding one-missing hypothesis then the test is positive. The feature composition of the pharmacophore, the value of correlation coefficient and the quality of the estimated activity of the left-out compound were used as measures for the assessment of the statistical test. By leaving each one of the 20 training set compounds according to this method, 20 new hypotheses were generated. As a result we did not obtain any meaningful differences between Hypo1 and each hypothesis resulting from the leave-one-out method. This result gives more confidence on Hypo 1 that it does not depend on one particular compound in the training set.

### Database screening

The validated pharmacophore hypothesis, Hypo1, was used as a 3D structural query for retrieving compounds from chemical databases including MayBridge (59 652 compounds), Chembridge (50 000 compounds), NCI (238 819 compounds) and Asinex (213 462 compounds). As a result of first screening 11 578, 590, 5096 and 63 265 compounds were retrieved from Maybridge, Chembridge, NCI and Asinex respectively. Since the active site of BACE-1 is larger in size, the experimentally known most active inhibitors are also larger in size and violate the first rule of Lipinski’s rule of five. Hence, the retrieved hit compounds were filtered based only on the estimated activity values calculated by Hypo 1. The activity range for the most active compounds is <100 nM. Finally 773 compounds were selected by restricting the minimum estimated activity to <100 nM.

### Molecular docking

**Figure 5:**
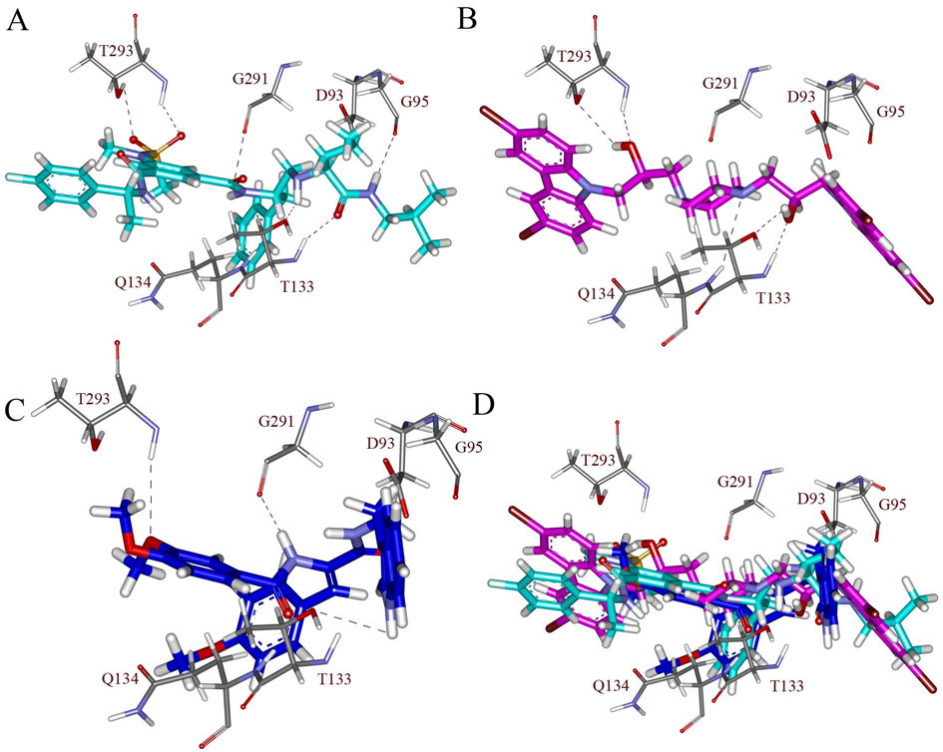
**Molecular docking results.** Binding orientations of A) Compound 1 of the training set (cyan color), B) RJC01726 (magenta color), C) Asnx-2 (blue color), D) overlay of Compound 1, RJC01726 and Asnx-2 in the active site of BACE-1 protein. Active site residues are shown in stick form and hydrogen bond interactions are indicated with purple dotted lines.

**Figure 6:**
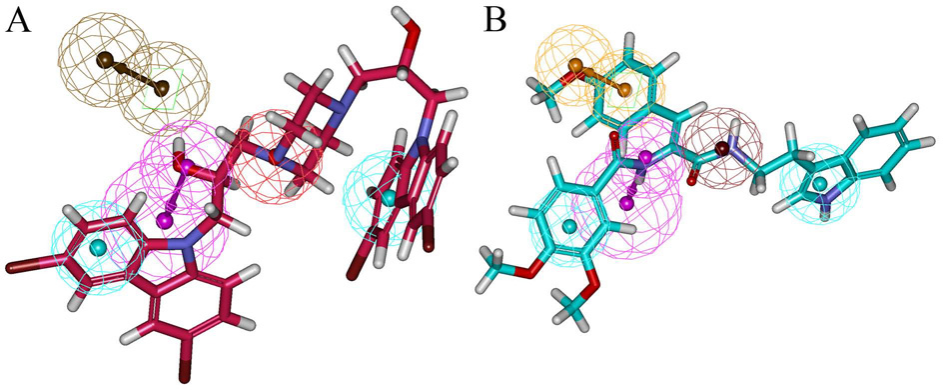
**Pharmacophore overlay on final hits.** The mapping of pharmacophore hypothesis Hypo 1 on the final hits. A) RJC01726 (red color) B) Asnx-2 (cyan color).

**Figure 7:**
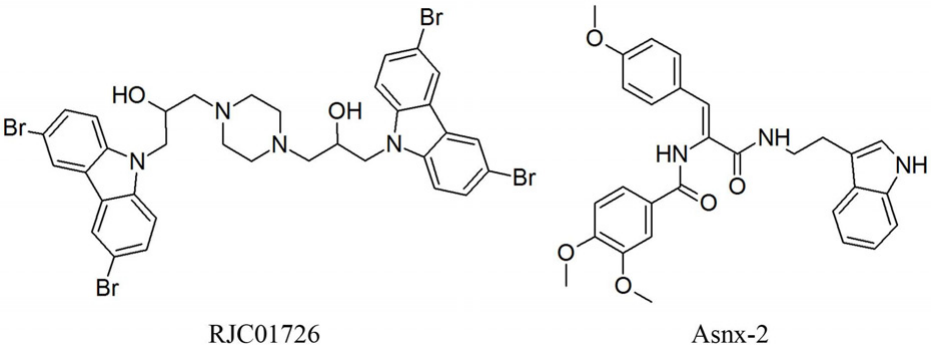
**Chemical structure of final hits.** 2D representation of the final hits RJC01726 and Asnx-2.

To further refine the retrieved hits and also to remove the false positives, these 773 compounds along with the 20 training set compounds were docked into the active site of BACE-1 using GOLD 4.1 program. There are number of crystal structures for BACE-ligand complexes are available in PDB. The crystal structure of BACE-SC7 (PDB ID: 2QP8) complex was taken based on its high resolution. The GOLD fitness score was calculated for all the 793 compounds, it distinguishes molecules based on their interacting ability. The GOLD fitness score for the most active compound in the training set was 53.035. The compounds for further analysis were selected based on the ligand conformations which can satisfy the necessary interactions at the active site and scoring GOLD fitness score greater than 60. Finally 20 compounds from Maybridge and 15 compounds from Asinex have shown the required interaction with BACE-1 as well as good GOLD fitness scores. The compounds with the same chemical scaffolds were filtered carefully based on the molecular interactions observed at the active site. Finally, two compounds with different scaffolds one from Maybridge (RJC01726) and one from Asinex (Asnx-2) were selected as representative compounds. The binding mode of the final hits and the most active Compound 1 in the training set are shown in Figure 5. Figure 5A represents the binding mode of Compound 1 with a GOLD fitness score of 53.035. It has formed hydrogen bond interactions with D93, G95, T133, Q134, G291 and T293 and hydrophobic interactions with Y132, F169, and T292. The GOLD fitness score of RJC01726 was 68.289 and the mode of binding in the active site (Figure 5B) is similar to Compound 1. It has formed hydrogen bond interactions with T133, Q134 and T293 and hydrophobic interactions with D93, G95, F169 and T292. Asnx-2 has shown hydrogen bond interactions with T133, G291 and T293 as well as hydrophobic interactions with D93, Y132, F169 and T292 with a GOLD fitness score of 62.026 (Figure 5C). Figure 5D represents the overlay of most active Compound 1, RJC01726 and Asnx-2 at their binding modes. The pharmacophore overlay of the final hits compounds are shown in Figure 6 and their 2D representations are shown in Figure 7.

The hydrophobic interactions of the final hits compounds were observed using *Ligplot* program [39]. The novelty of the two hits compounds were confirmed using S*ciFinder* search [40] and *PubChem* search [41].

## Conclusion

A chemical feature based 3D pharmacophore hypotheses of BACE-1 inhibitors have been developed using *3D QSAR Pharmacophore Generation* protocol available in DS 2.5. The best quantitative pharmacophore model, Hypo 1, was characterized by the highest cost difference (121.98), best correlation coefficient (0.977), lowest total cost value (81.24) and lowest RMSD (0.804). The fixed cost and null cost values were 74.77 and 203.22 bits, respectively. Hypo1 consisted of one HBD, one PI, one RA and two HY features. Hypo1 was further validated by test set, Fischer randomization test, leave-one-out, and decoy set methods. The test set containing 40 compounds was used in investigating the predictive ability of Hypo1 and resulted with a correlation coefficient of 0.917. Other validation methods also have provided reliable results on the strength of Hypo 1. This validated Hypo1 was used as a 3D query in database screening. The database hit compounds were subsequently subjected to filtering by estimated activity value. To further refine the retrieved hits the 793 compounds along with training set were carried out for molecular docking studies. The molecular docking result of all compounds was analyzed based on the GOLD fitness score, binding modes and molecular interactions with essential active site residues. Finally, two hits, namely, RJC01726 and Asnx-2 of different scaffolds with GOLD fitness score of 68.362 and 63.053, respectively, and interactions with important active site residues were chosen as lead candidates. These compounds as such and on further optimization can be used as potential leads in designing new BACE-1 inhibitors.

## Competing interests

The authors declare that they have no competing interests.

## Author’s contributions

SJ and ST equally involved in designing the work, analyzing the results and writing the manuscript. SS formatted and corrected the manuscript. KWL supervised the work and edited the manuscript. All four authors have read and approved the manuscript.

## Supplementary Material

Additional file 1 Experimental and estimated IC50 values of the test set compounds based on the pharmacophore hypothesis ‘Hypo 1’.). Click here for file
