# Supplementary material for: Potent bace-1 inhibitor design using pharmacophore modeling, in silico screening and molecular docking studies
Source: BMC Bioinformatics. 2011 Feb 15;12(Suppl 1):S28. doi: 10.1186/1471-2105-12-S1-S28 (PMC3044283; doi:10.1186/1471-2105-12-S1-S28)
Supplement: Additional file 1 — Experimental and estimated IC50 values of the test set compounds based on the pharmacophore hypothesis ‘Hypo 1’.). [file 1471-2105-12-S1-S28-S1.doc]

**Table 3:** **Experimental and estimated IC_50_ values of the test set compounds based on the pharmacophore hypothesis ‘Hypo 1’**.

| Compound | IC_50_ nM | | Error^a^ | Activity scale^b^ | |
| --- | --- | --- | --- | --- | --- |
|  | Experimental | Estimated |  | Experimental | Estimated |
| 21 | 8 | 10.73 | +1.3 | ++++ | ++++ |
| 22 | 20 | 30.41 | +1.5 | ++++ | ++++ |
| 23 | 24 | 28.13 | +1.2 | ++++ | ++++ |
| 24 | 27 | 68.77 | +2.5 | ++++ | ++++ |
| 25 | 32 | 55.86 | +1.7 | ++++ | ++++ |
| 26 | 35 | 73.88 | +2.1 | ++++ | ++++ |
| 27 | 110 | 801.74 | +7.3 | +++ | +++ |
| 28 | 110 | 150.55 | +1.4 | +++ | +++ |
| 29 | 115 | 320.76 | +2.8 | +++ | +++ |
| 30 | 117 | 523.53 | +4.5 | +++ | +++ |
| 31 | 139 | 102.54 | -1.4 | +++ | +++ |
| 32 | 180 | 479.79 | +2.7 | +++ | +++ |
| 33 | 223 | 712.92 | +3.2 | +++ | +++ |
| 34 | 317 | 108.27 | -2.9 | +++ | +++ |
| 35 | 418 | 997.71 | +2.4 | +++ | +++ |
| 36 | 420 | 471.57 | +1.1 | +++ | +++ |
| 37 | 430 | 760.89 | +1.8 | +++ | +++ |
| 38 | 630 | 156.61 | -4.0 | +++ | +++ |
| 39 | 1100 | 109.39 | -10.0 | ++ | +++ |
| 40 | 1300 | 2033.21 | +1.6 | ++ | ++ |
| 41 | 1370 | 8862.94 | +6.5 | ++ | ++ |
| 42 | 1400 | 1935.82 | +1.4 | ++ | ++ |
| 43 | 1400 | 7905.24 | +5.6 | ++ | ++ |
| 44 | 2700 | 3027.39 | +1.1 | ++ | ++ |
| 45 | 2900 | 8593.14 | +3.0 | ++ | ++ |
| 46 | 3400 | 5371.09 | +1.6 | ++ | ++ |
| 47 | 4630 | 9939.21 | +2.1 | ++ | ++ |
| 48 | 5500 | 33347.74 | +6.0 | ++ | + |
| 49 | 5900 | 1658.48 | -3.6 | ++ | ++ |
| 50 | 5900 | 12466.43 | +2.1 | ++ | + |
| 51 | 9900 | 25021.51 | +2.5 | ++ | + |
| 52 | 11000 | 1741.43 | -6.3 | + | ++ |
| 53 | 15000 | 20515.4 | +1.4 | + | + |
| 54 | 16000 | 12596.9 | -1.3 | + | + |
| 55 | 17200 | 37380.6 | +2.2 | + | + |
| 56 | 21000 | 49617.2 | +2.4 | + | + |
| 57 | 21000 | 10021.6 | -2.1 | + | + |
| 58 | 22000 | 11300.8 | -2.0 | + | + |
| 59 | 24000 | 33848.8 | +1.4 | + | + |
| 60 | 30000 | 58292.7 | +1.9 | + | + |

^a^Positive value indicates that the estimated IC_50_ is higher than the experimental IC_50_; negative value indicates that the estimated IC_50_ is lower than the experimental IC_50_.

^b^Activity scale: most active, ++++, IC_50_ ≤ 100 nM; active, +++, 100 nM < IC_50_ ≤ 1000 nM; moderately active, ++, 1000 nM < IC_50_ ≤ 10,000 nM; inactive, +, IC_50_ > 10,000 nM.
